# The Sphinx’s riddle: cardiovascular involvement in autoimmune rheumatic disease

**DOI:** 10.1186/s12872-016-0381-5

**Published:** 2016-10-28

**Authors:** Sophie Mavrogeni, George Markousis-Mavrogenis, Genovefa Kolovou

**Affiliations:** Onassis Cardiac Surgery Center, 50 Esperou Street, 175-61 P. Faliro, Athens Greece

**Keywords:** Rheumatic diseases, Cardiovascular magnetic resonance imaging

## Abstract

Factors leading to Cardiovascular Disease (CVD) in Autoimmune Rheumatic Diseases (ARD) include: a) atherosclerosis and macro-microvascular coronary artery disease b) pericardial, myocardial and vascular inflammation c) heart valve disease d) heart failure and e) pulmonary hypertension.

Cardiology utilizes various non-invasive imaging modalities, such as rest/stress Electrocardiogram (ECG), echocardiography, nuclear imaging and more recently Cardiovascular Magnetic Resonance (CMR) to detect ischemic or inflammatory disease in ARD. Exercise ECG is a reliable prognostic test for identification of patients either very unlikely or very likely to have cardiac events. However, this is not the case for intermediate risk patients. In stress echocardiography the diagnostic end point for the detection of myocardial ischemia is the induction of a transient worsening in regional function during stress. It provides similar diagnostic and prognostic accuracy as radionuclide stress perfusion, but at a lower cost and without radiation exposure. Stress Myocardial Perfusion Scintigraphy (MPS) is a non-invasive imaging modality for patients with suspected coronary artery disease, but has important limitations including radiation exposure, imaging artefacts and low spatial resolution, which preclude detection of small myocardial scars commonly found in ARD. By identifying early stages of inflammation and perfusion defects, CMR can shed light on the exact pathophysiologic background of myocardial lesions, even if the underlying ARD seems stable. However, high cost and lack of availability and expertise limit wider adoption.

Hopefully, CMR will not have the same fate as Oedipous, who despite answering the Sphinx’s riddle successfully, finally came to a bitter end; for in the case of CMR overcoming fate is, in fact, in our hands.

## Background

In Greek mythology, the Sphinx was a monster with the head of a woman, the wings of a griffin and the body of a lion. The Sphinx stopped travellers on the road to the city of Thebes and asked them a riddle. If they answered wrong they were killed by the Sphinx. Cardiovascular Disease (CVD) is the Sphinx of our times in the management of Autoimmune Rheumatic Diseases (ARD) and the riddle, for which we also seem to lack the answer, is a complex cardiovascular pathophysiology. The systemic manifestations of ARD attract most of the attention of attending rheumatologists, while cardiologists are mostly unaware of CVD in ARD. Lack of diagnostic algorithms for early assessment of ARD, when CVD is still silent only compounds these issues [1]. Furthermore, although use of targeted treatment in ARD has led to a reduction of disease-associated mortality, increased CVD incidence lowers life expectancy compared to the general population [2–7].

The clinical scenarios leading to increased CVD in ARD include:

- Atherosclerosis and macro-microvascular Coronary Artery Disease (CAD). These are the commonest suspects causing increased CVD and mortality in ARD [8]. Rheumatoid Arthritis (RA) has a 1.5- to 2.0-fold risk of CAD, compared with controls, even before the diagnosis of arthritis [9–11]. Traditional risk assessment usually underestimates CVD risk in ARD [12, 13]. Furthermore, inflammation and autoimmunity, the main characteristics of ARD, promote the development of atherosclerosis [14, 15]. RA, spondyloarthropathies, Systemic Lupus Erythematosus (SLE), Antiphospholipid Syndrome (APS) and systemic vasculitides have all been associated with accelerated atherosclerosis [16–20, 38]. Notably, CAD risk in RA is similar to that of diabetes mellitus and the associated atherosclerotic plaques are more susceptible to instability and rupture, with higher re-infraction rates and worse prognosis [21–23].
  Although SLE presents with less “inflammatory reaction” compared with RA and/or spondyloarthropathies, it is characterized by higher incidence of CVD. Atherosclerosis in SLE is associated with older age at diagnosis and longer disease duration, supporting the hypothesis that chronic exposure to SLE immune dysregulation promotes CVD [24]. Early damage of both macro- and microvasculature occurs before or shortly after SLE diagnosis [25, 26]. Macro-or microvascular CAD is also common in SLE, with 54 % of patients having non-calcified coronary plaques and impaired coronary flow reserve, even in patients with seemingly normal coronary arteries [27, 28]. These findings are well correlated with disease activity and disease duration [27, 29–33]. Finally, High Density Lipoprotein (HDL) is decreased in SLE, while Low Density Lipoprotein (LDL), Very Low Density Lipoprotein (VLDL), proinflammatory HDL and triglyceride levels are increased [34, 35]. Therefore, early treatment of immune dysregulation is imperative for CAD prevention.
- Pericardial, Myocardial and Vascular inflammation. Inflammatory heart disease, despite being considered a rare complication of ARD, can significantly contribute to increases in CVD mortality. The typical appearance of myopericarditis is more common in SLE, but also has notable impact in CVD morbidity of other ARDs, such as RA, mixed collagen diseases, systemic sclerosis, vasculitis, sarcoidosis and inflammatory myopathies [35]. Overt clinical presentation of myopericarditis is typically found only in a minority of SLE patients. In the majority of ARD patients, however, myopericarditis remains silent, leading to delays in diagnosis which in turn can cause deterioration into Heart Failure (HF) and arrhythmias [36].
  Additionally, inflammation of large-, medium- and small-sized blood vessel walls, either due to primary or secondary ARD, may lead to stroke, myocardial infarction, visual abnormalities, limb/jaw claudication and digital ulcers [37]. Myocardial vasculitis is common in medium/small vessel vasculitis and in APS; it may cause myocardial ischemia due to involvement of epicardial coronary arteries, or small intramural cardiac vessels, finally leading to Left Ventricular (LV) impairment [38, 39].
- Heart Valve Disease (HVD). HVD is the commonest cardiac disease in APS, with a prevalence of 30 %. Diagnosis is based on the presence of valvular thickening or vegetations (mainly mitral and aortic) as described by Libman and Sacks for patients with SLE [40]. The coexistence of Antiphospholipid Antibodies (aPL) with SLE carries a 3-fold greater risk of HVD and confirms that the involvement of these antibodies in the pathophysiologic mechanism leads to valve thrombosis secondary to hypercoagulability [41]. Progressive valve disease has also been identified in RA [42, 43]. Transthoracic and/or Transoesophageal Echocardiography (TTE and TEE, respectively) are currently the cornerstone of early and accurate diagnosis [41].
- Heart Failure (HF). HF in ARD can be caused by ischemic or non-ischemic heart disease and contributes to increased mortality [44]. In RA, the prevalence of HF is 2-fold higher than in general population and remains increased after adjusting for classic CVD risk factors and ischemic heart disease [45, 46]. HF is also seen in SLE, inflammatory myopathies, systemic sclerosis, ankylosing spondylitis and vasculitis, as the endpoint of myocardial inflammation [47–50].
  Fibrotic cardiomyopathy, highly prevalent in systemic sclerosis, vasculitis, myositis and sarcoidosis, may also lead to impaired LV function [51]. However, LV systolic function remains normal until the late stages of the disease and only imaging techniques such as Tissue Doppler and Cardiovascular Magnetic Resonance (CMR) can identify these lesions at a preclinical stage [52, 53].
- Pulmonary Arterial Hypertension (PAH). PAH affects 0.5-15 % of ARD and carries worse prognosis compared with idiopathic PAH. It is most common in systemic sclerosis and is responsible for 30 % of disease-related deaths [54]. Microangiopathy and chronic hypoxemia secondary to interstitial lung fibrosis are the major causes of PAH in SSc [55]. LV dysfunction and thromboembolic disease are additional causes of PAH in ARD and are due to APS and primary Sjogren syndrome respectively [56, 57]. Despite the use of new imaging modalities, more than 50 % of PAH in ARD is missed, and is diagnosed only when the disease is in advanced stages [58]. A schematic expression of various clinical scenarios of CVD in ARD was presented in Table 1.

**Table 1:** Clinical scenarios of CVD in ARD

Clinical scenarios	Coronary artery disease	Micro-vascular disease	Myocarditis/Pericarditis/Vasculitis	PAH	HF	Valve disease
Typical chest pain	++++	++	+++	+	+	+
Atypical chest pain	++	+++++	+++	++	++	+
Shortness of breath	++	+++	++++	++++++	++++	++++
Syncope	+	++	++	++++	+++	++++
Arrhythmia	++	++	+++	++	++++	++++

## Cardiovascular diagnosis in the era of multimodality imaging

Cardiology uses various non-invasive imaging modalities, such as rest/stress Electrocardiogram (ECG), echocardiography, nuclear techniques and more recently CMR, to detect ischemic or inflammatory disease in ARD. Exercise ECG (ExECG) is a reliable prognostic test for identification of patients who are either very unlikely or very likely to have cardiac events. However, this is not the case for intermediate risk patients or patients with small vessel vasculitis, who may be missed both by routine non-invasive as well as invasive assessment [59]. ECGs can be indicative of myocarditis, and there is an association between transmural LV myocardial oedema detected by CMR and T wave inversion in clinically suspected acute myocarditis. However, this is an expression of reversible myocardial oedema during the acute disease phase and can not be used as a predictor of LV systolic dysfunction during follow-up [60].

Echocardiography is the cornerstone imaging technique to assess cardiac morphology in everyday practice. The addition of new techniques, such as speckle tracking echocardiography and in particular longitudinal deformation, has been successfully used for the assessment of subclinical LV dysfunction and monitoring of the effects of anakinra, an interleukin-1 receptor antagonistant, in treatment on LV function in ARD patients, particularly when CAD coexists [61–64].

Stress echocardiography is defined as a combination of 2D echocardiography either with a physical or pharmacological stress. The diagnostic end point for the detection of myocardial ischemia is the induction of a transient worsening in regional function during stress. It has similar diagnostic and prognostic accuracy as radionuclide stress perfusion tests, but at a lower cost, without environmental impact and with no biohazards for patients and physicians. Additionally, the assessment of coronary flow reserve by Doppler echocardiography can reveal microvascular disease in ARD [65] and also subclinical coronary artery disease [66]. Despite being an operator dependent technique, unable to perform tissue characterisation, echocardiography is the most cost-effective approach for the non-invasive exclusion and/or evaluation of the CAD possibility [67].

Stress Myocardial Perfusion Scintigraphy (MPS) is a non-invasive imaging modality used in patients with suspected CAD, commonly found in RA and SLE [68]. However, MPS has serious limitations including radiation exposure, imaging artefacts and low spatial resolution, which do not permit detection of subendocardial ischemia and small scars seen in ARD [69]. The CE-MARC study currently supports wider adoption of CMR for CAD assessment, owing to concerns regarding carcinogenesis risks associated with medical use of ionising radiation [70]. In a study comparing of Single Photon Emission Tomography (SPECT), Positron Emission Tomography (PET), and CMR, all techniques were shown to have high sensitivity, with a broad range for specificity observed in CMR [71].

## Conclusions

To correlate Greek mythology with more contemporary terms, CMR now takes the place of Oedipus in our paradigm. Like its “predecessor”, CMR is capable of successfully answering the riddle of CVD in ARD, by increasing our diagnostic capabilities and detecting cardiac pathology before any change in systolic or diastolic indexes takes place [1]. Additionally, by identifying early stages of inflammation and perfusion defects, CMR can shed light on the exact pathophysiologic background of myocardial lesions even if the underlying ARD appears stable. It also provides the additive value of being able to assess myocardial status independently of systemic inflammatory processes, leading to individualization of cardiac treatment, irrespective of ARD status [1]. Finally, stress perfusion CMR allows for early detection of microvascular disease, a common finding in ARD, commonly missed by nuclear imaging techniques due to their low spatial resolution [69].

Unfortunately, lack of availability and expertise, insufficient collaboration between cardiologists and radiologists, unfamiliarity of rheumatologists with the advantages of CMR in ARD and significant associated costs, especially when a stress study is added, are serious obstacles preventing more widespread adoption of CMR in ARD monitoring. Hopefully, this excellent diagnostic tool will not have the same fate as Oedipous, who despite successfully answering the Sphinx’s riddle, finally came to a bitter end; for in the case of CMR, overcoming fate is, in fact, in our hands.
